# Serum Antioxidant Associations with Metabolic Characteristics in Metabolically Healthy and Unhealthy Adolescents with Severe Obesity: An Observational Study

**DOI:** 10.3390/nu10020150

**Published:** 2018-01-30

**Authors:** Ana Paula Stenzel, Roberta Carvalho, Patricia Jesus, Aline Bull, Silvia Pereira, Carlos Saboya, Andrea Ramalho

**Affiliations:** 1 School of Medicine, Federal University of Rio de Janeiro (UFRJ), Rio de Janeiro 21.941-902, Brazil; stenzel.ap@gmail.com (A.P.S.); pcj.nutricao@gmail.com (P.J.); 2 Center for Research on Micronutrients (NPqM), Institute of Nutrition Josué de Castro of UFRJ, Rio de Janeiro 21.941-902, Brazil; alinebull@yahoo.com.br (A.B.); se.pereira@gmail.com (S.P.); cjsaboya@carlossaboya.com.br (C.S.); aramalho.rj@gmail.com (A.R.); 3 Multidisciplinary Center for Bariatric and Metabolic Surgery, Rio de Janeiro 22.280-020, Brazil; 4 Department of Social and Applied Nutrition of the Institute of Nutrition, UFRJ, Rio de Janeiro 21.941-902, Brazil; 5 Escola Paulista de Medicina, Federal University of São Paulo (UNIFESP), São Paulo 04.021-001, Brazil

**Keywords:** obesity, adolescents, Metabolically Healthy Obesity, antioxidants

## Abstract

Considering the inadequacy of some antioxidant nutrients in severely obese adolescents, this study aimed to assess the relationship between antioxidant micronutrients status and metabolic syndrome components in metabolically healthy obesity (MHO) and unhealthy obesity (MUO). We performed an observational study in severely obese adolescents (body mass index > 99th percentile) and they were classified into MHO or MUO, according to the criteria adapted for adolescents. Anthropometric, biochemical, and clinical variables were analyzed to characterize the sample of adolescents. The serum antioxidant nutrients assessed were retinol, β-carotene, Vitamin E, Vitamin C, zinc and selenium. A total of 60 adolescents aged 17.31 ± 1.34 years were enrolled. MHO was identified in 23.3% of adolescents. The MHO group showed lower frequency of non-alcoholic fatty liver disease (14.3% vs. 78.3%, *p* < 0.001) when compared to MUO. A correlation was found between retinol and β-carotene concentrations with glycemia (*r* = −0.372; *p* = 0.011 and *r* = −0.314; *p* = 0.034, respectively) and between Vitamin E with waist circumference (*r* = −0.306; *p* = 0.038) in the MUO group. The current study shows that some antioxidant nutrients status, specifically retinol, β-carotene, and Vitamin E, are negatively associated with metabolic alterations in MUO. Further studies are necessary to determine the existing differences in the serum antioxidant profile of metabolically healthy and unhealthy obese adolescents.

## 1. Introduction

Childhood obesity and its associated metabolic complications is rapidly emerging worldwide [1]. The degree of pediatric obesity is increasing and will likely have profound impact on adult morbid obesity and other morbidities [2], reducing life quality and expectancy.

Although obesity is an important risk factor for the development of several metabolic complications that can increase the risk of cardiovascular diseases (CVD), some studies have shown the existence of a subgroup of individuals with obesity that appear to be better protected from these complications [3,4]. These individuals have a metabolically healthy obesity (MHO) and, despite presenting excess body fat, have a less compromised metabolic profile [4].

Different criteria to identify MHO have been adopted and the one mostly used in clinical practice is that used for diagnosing metabolic syndrome (MS), proposed by the Third Report of the National Cholesterol Education Program Expert Panel on Detection, Evaluation, and Treatment of High Blood Cholesterol in Adults (NCEP ATP III) [3,4].

Although not included as one of the available criteria for the identification of individuals with MHO, non-alcoholic fatty liver disease (NAFLD) has been investigated in these individuals due to its associations with obesity. Evidence in the literature has shown that individuals with MHO have less hepatic fatty accumulation when compared to individuals with metabolically unhealthy obesity (MUO) [5], which is related to increased cardiometabolic risks [6].

It is interesting to assess associations between obesity metabolic abnormalities and reduction of serum concentrations of antioxidant nutrients. The literature has shown that obese children can have lower concentrations of serum antioxidant nutrients. These micronutrients are involved in important metabolic and endocrine processes related to genesis and control of overweight, like Vitamins A, E, and C and the minerals zinc and selenium [7]. However, the relationship between these micronutrients’ status and metabolic abnormalities in adolescents with MHO and MUO has not been previously investigated.

Given this, considering the increased prevalence of severe obesity in adolescents and the clinical importance of cardiometabolic changes related to the identification of MHO, the current study aimed to evaluate the relationship between antioxidant micronutrients status and metabolic syndrome components in obese adolescents according to the MHO and MUO condition, classified according to the NCEP ATP III criteria.

## 2. Materials and Methods

### 2.1. Participants

Adolescents were considered if aged 10–19 years and 11 months with body mass index (BMI)/age over the 99th percentile for age and gender [8]. Exclusion criteria were: prior disabsorptive and restrictive surgeries, disabsorptive intestinal syndromes, neoplasias, use of lipid-lowering drugs, use of hypoglycemic medications for weight loss and vitamin and mineral supplements, pregnant women, nursing mothers, and kidney and liver diseases, except for NAFLD.

This study was approved by the Research Ethics Committee of the Hospital Universitário Clementino Fraga Filho (HUCFF) (University Hospital Clementino Fraga Filho) at the Federal University of Rio de Janeiro under the ethical code 011/10 in accordance with resolution No. 196 of the Conselho Nacional de Saúde (National Health Council). Informed consent and assents were obtained from patients or parents.

### 2.2. Study Design

This is an observational study, with adolescents with severe obesity as participants [8], attended in a multidisciplinary center for control of obesity in Rio de Janeiro city. Data collection was conducted from 2013 to 2015.

To classify metabolically healthy obesity, the criteria proposed by the NCEP ATP III [9] were used and adapted to adolescent individuals [10]. Such criteria adopted was analogous to ATP III as ≥3 of the following: (1) fasting triglycerides ≥ 100 mg/dL; (2) High Density Lipoprotein (HDL) < 50 mg/dL, excepted in boys aged 15 to 19 years, in whom the cut-off point was <45 mg/dL; (3) fasting glucose ≥ 110 mg/dL; (4) waist circumference (WC) > 75th percentile for age and gender; and (5) systolic blood pressure > 90th percentile for gender, age, and height.

### 2.3. Measurements

Anthropometric, clinical, and biochemical assessments were available in order to characterize the sample of obese adolescents and to analyze the associations of the serum antioxidant nutrients status with the metabolic variables in MHO and MUO adolescents.

In the anthropometric assessment, body weight and height were measured to calculate BMI. WC was also obtained. All measurements were taken in duplicate by a single trained evaluator with 0.5 cm variations accepted and mean value was calculated.

Clinical variables were determined by NAFLD and systemic arterial hypertension (SAH) frequencies.

NAFLD diagnosis was determined by a total abdomen ultrasound (US) device Philips Medical Systems Ltda, Visor C and 2–5 MHz (Barueri, São Paulo, Brazil). The presence of NAFLD was diagnosed by a single specialist physician in diagnostic imaging.

SAH diagnosis was conducted by a specialized professional, as proposed by the VI Brazilian Guideline of Arterial Hypertension (2010) [11]. Data on the diagnoses of SAH and NAFLD were obtained through the patient’s medical records.

Samples of fasting blood were collected for biochemical assessments. Glycemia was obtained by the enzymatic colorimetric method. Insulin resistance was estimated by the Homeostasis Model Assessment-Insulin Resistance (HOMA-IR) formula. Serum concentrations of total cholesterol and triglycerides were analyzed by the enzymatic colorimetric method, and fractional Low Density Lipoprotein (LDL-c) and HDL-c were obtained by the selective inhibition method. C-reactive protein (CRP) was quantified by the nephelometry method.

Serum vitamins were quantified by High Performance Liquid Chromatography with Ultraviolet Detector (HPLC-UV) (Labtest Diagnóstica S.A., Lagoa Santa, Minas Gerais, Brazil). Nutritional status of Vitamin A was performed by the quantification of serum concentrations of retinol and β-carotene, and the cut-off points for inadequacy were <1.05 µmol/L and ≤40 µg/dL, respectively [12,13]. The cut-off points of Vitamins C and E were <4.6 mg/L and <0.5 mg/dL, respectively [14].

Serum minerals zinc and selenium were quantified by Atomic Absorption Spectrophotometry (Thermo Scientific, Waltham, MA, USA) with the cut-off points <75 µg/L and <70 µg/dL, respectively [15].

### 2.4. Statistical Analyses

Statistical analyses were conducted using the Statistical Package for the Social Sciences (SPSS) for Windows version 22.0, IBM Corporation (Armonk, NY, USA). The Mann Whitney U and Chi-square tests were applied to assess the characteristics of the sample adolescents according to groups with MHO and MUO. The Spearman test was used to evaluate correlations between each antioxidant micronutrient with each diagnostic criterion for MS proposed by the NCEP ATP III regarding groups with MHO and MUO. The significance level adopted was 5% (*p* < 0.05).

## 3. Results

The sample consisted of 60 adolescents with severe obesity, mean age of 17.31 ± 1.34 years, BMI of 46.28 ± 7.20 kg/m^2^, and 63.3% of them were female. No statistically significant difference was found between genders among the variables analyzed. The classification of adolescents in metabolically healthy and unhealthy, according to the criteria recommended by the NCEP ATP III, showed a percentage of 23.3% of participants with MHO.

Table 1 shows cardiometabolic variables of the obese adolescents of the present study according to classification as MHO and MUO. Serum concentrations of total cholesterol and CRP were elevated. The proportion of participants who had inadequacy of these parameters status over 80% were found both in patients with MHO and in patients with MUO without significant changes between them (data not shown).

We observed that the prevalence of NAFLD was significantly higher in MUO when compared to MHO with a Relative Risk (RR) of 21.6 (95% CI, 5.25–94.6 *p* < 0.001), meaning that adolescents with MUO are 21 times more likely to have NAFLD than MHO. Prevalence of SAH was also significantly higher in MUO when compared to MHO besides being an inclusion criterion of the NCEP ATP III. In the same way, HDL-c (45.1 ± 10.0 mg/dL vs. 51.9 ± 7.7 mg/dL, *p* = 0.022) was significantly lower in MUO whereas triglycerides concentrations (135.3 ± 50.4 mg/dL vs. 104.2 ± 37.3 mg/dL, *p* = 0.028) were significantly higher in MUO when compared to MHO.

Regarding the proportion of participants who had inadequate micronutrients status with antioxidant function analyzed in the overall sample, we observed 91.7% of deficiency of Vitamin C and 66.7% of β-carotene deficiency (Table 2).

**Figure 1:**
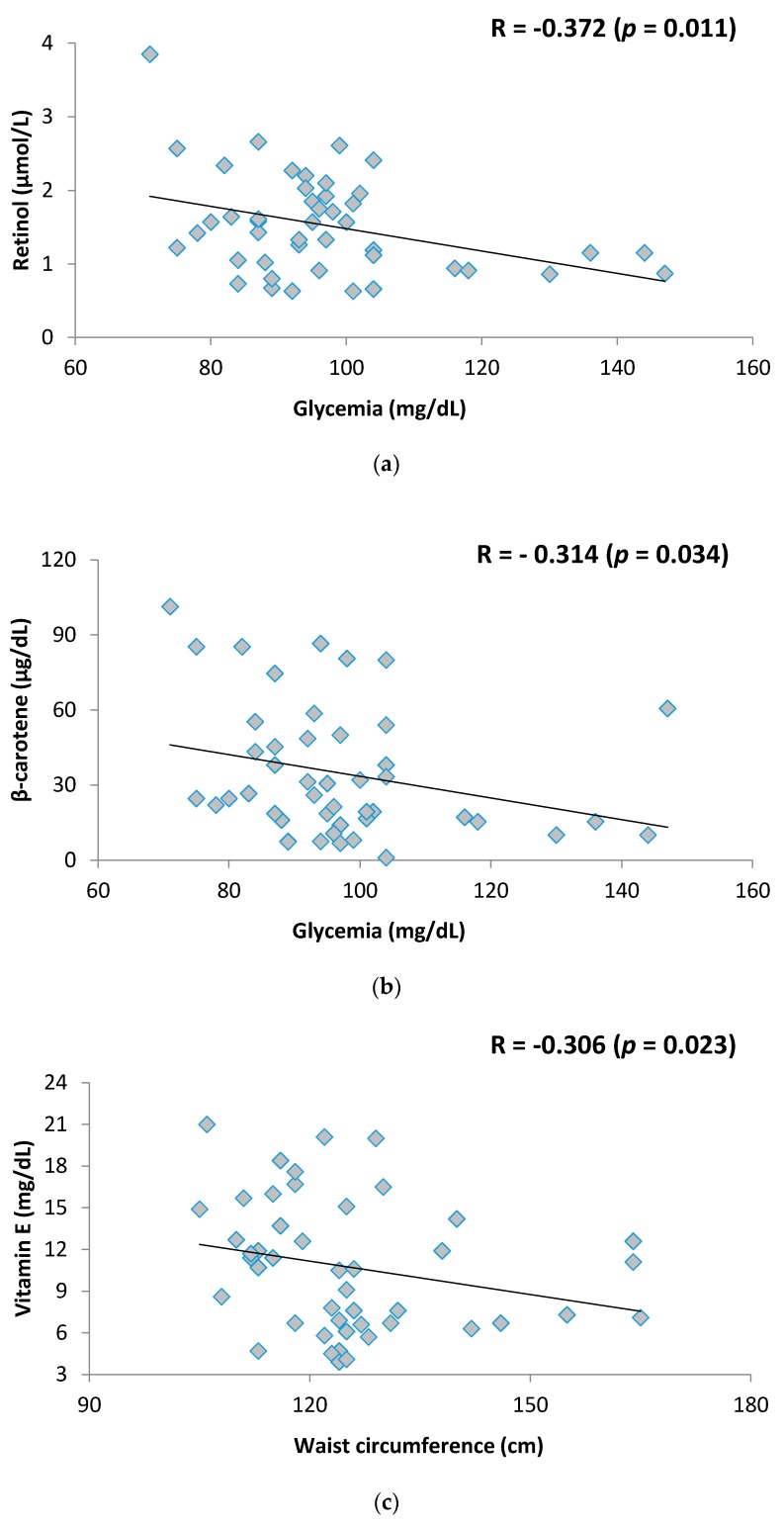
(**a**) Association between retinol and glycemia in metabolically unhealthy obese (MUO) adolescents; (**b**) Association between β-carotene and glycemia in MUO adolescents; (**c**) Association between Vitamin E and waist circumference (WC) in MUO adolescents.

When associations between each antioxidant micronutrient with MS components were conducted in groups MHO and MUO, only the MUO group showed a significant correlation between retinol and glycemia (*r* = −0.372; *p* = 0.011), β-carotene and glycemia (*r* = −0.314; *p* = 0.034), and Vitamin E and WC (*r* = −0.306; *p* = 0.038) (Figure 1).

## 4. Discussion

The results of the present study show that some antioxidant nutrients status, specifically retinol, β-carotene, and Vitamin E, are negatively associated with metabolic alterations in MUO adolescents. This work explored the antioxidant nutrients status in adolescents with MHO and MUO. Adolescence is a stage of life with increased nutritional demands for growth and hormonal changes, and there is much evidence assuming that obese individuals with MUO are more prone to subclinical inflammation than obese individuals without this metabolic abnormalities [16]. In this way, adolescents with severe obesity require greater care since they have several metabolic conditions that contribute to the inflammatory status and, consequently, to oxidative stress, increasing the use of antioxidant micronutrients.

The NCEP ATP III diagnostic criterion variable that presented most significant alteration in MUO when compared to MHO was SAH. Prince and co-workers (2014) [3], also found a significant higher prevalence of individuals with MUO with SAH, when compared to MHO. These results deserve special attention because previous evidence has established that changes in blood pressure tend to remain and escalate into adulthood [17].

NAFLD was more prevalent in obese with MUO than MHO. Even though this disease is not part of the metabolic changes included in the NCEP ATP III classification criteria, this condition seems to be significantly lower in individuals with MHO when compared to individuals with MUO [5]. Although the individuals with MUO did not have increased HOMA-IR measures, contrasting expectations, this could be explained as reported in animals’ studies [18,19] that a decreased triglyceride accumulation in the liver could occur with an increased triglyceride release into the bloodstream. This shows that although NAFLD is not present in obese adolescents, this absence does not exclude them from having insulin resistance at some future stage in life.

Despite the high proportion of participants who had elevated serum concentrations of LDL-c in the total sample, its inadequacy was higher in participants with MUO.

We have observed that almost all of the adolescents assessed showed changes in LDL-c and CRP, regardless of whether they had MHO or MUO. It is important to identify the presence of these abnormalities in such early stage of life, especially because LDL-c oxidation increases the risk of atherosclerotic disease [20], and CRP is an important marker of vascular inflammation and is, therefore, associated with CVDs [21]. There are few studies that include CRP for identification of individuals with MHO and MUO [22] since severe obesity is a potential inflammatory condition per se independent of metabolic alterations.

Regarding the assessment of the nutritional status of micronutrients with antioxidant function, we have found high proportion of participants who had low serum concentrations of serum Vitamin C. Garcia and co-workers (2013) [23] have shown that low serum concentrations of Vitamin C are associated with increased body fat and abdominal fat. A possible mechanism for this association could be due to ascorbic acid modulation on lipolysis in the interior of adipocytes and inflammatory response [24].

We have observed a higher proportion of participants who had inadequate β-carotene status in comparison to retinol status, which corroborates a finding in a previous study [25]. This finding could be explained because β-carotene has been diverted from its antioxidant functions for bioconversion to retinol, since it is important to preserve the serum concentrations of retinol and β-carotene is the most important precursor of retinol.

We have noted a higher proportion of participants with a higher inadequacy of retinol in serum when compared to a previous study conducted in adolescents, but those studies were conducted in adolescents with lower BMI percentiles [26]. The involvement of Vitamin A in body adiposity has been much addressed, and its contribution in the metabolic regulation of adipose tissue has been increasingly recognized [27]. Additionally, it is worth noting the action of retinol to maintain antioxidant defenses in situations of oxidative stress stimulus, as observed in increased body adiposity.

The study shows a negative correlation between Vitamin E and waist circumference, similar to other studies [28]. The relationship between this vitamin and increased adiposity has been investigated in the literature because of its functions in reducing adipose tissue fibrosis, inflammation, and oxidative stress due to its anti-inflammatory and antioxidant capacity.

The significant negative correlation between retinol and glucose observed in our study is similar to the findings of Teske and co-workers (2014) [26] in a study conducted with children and adolescents with obesity. Hyperglycemia is one of the conditions of obesity that contributes to oxidative stress, favoring the formation of reactive oxygen species, which increases the expense of micronutrients with antioxidant function [28]. In addition, since it is a condition related to type 2 diabetes mellitus, and to insulin resistance, there is evidence that retinol can increase insulin sensitivity by promoting its increased signaling [29].

A limitation of our study is its small sample size, which decreases the power to detect small differences, if any, between serum antioxidant nutrient status in group MHO and MUO. However, our sample is restricted to adolescents with severe obesity. We used abdominal US to detect NAFLD presence, although biopsy is a method more appropriate to identify and to measure the disease stage. However, considering the mean age of the sample of our study and that NAFLD is a progressive condition with later alterations, this aspect could be overcome. The study was also limited by having no dietary information about these individuals, since diet quality is a strong predictor of micronutrient status. It is presumed that although food consumption is excessive in kilocalories, it is not accompanied by adequate consumption of vitamins and minerals. Despite these limitations, the current study should help in the design of further studies exploring antioxidant status differences between obese adolescents with or without MS.

## 5. Conclusions

In conclusion, our data suggest that metabolically unhealthy obese adolescents have a significant inverse association between Vitamin A status and glycemia and between serum Vitamin E and waist circumference. Finally, future larger observational studies are needed to assess the impairment in nutritional status caused by metabolic abnormalities in obese adolescents and which nutrients should be investigated in the clinical care of these patients.

## Figures and Tables

**Table 1:** Anthropometric, clinical and biochemical variables in adolescents classified as having metabolically healthy or unhealthy obesity.

General Characteristics	MHO (*n* = 14)	MUO (*n* = 46)	*p*-Value
**Anthropometric Variables**			
BMI-kg/m^2^	45.4 ± 4.4	46.5 ± 8.0	0.740
WC-cm	120.4 ± 12.0	125.4 ± 14.6	0.217
**Clinical Variables**			
SAH-%	28.6	76.1	0.001 *
NAFLD-%	14.3	78.3	<0.001 *
**Biochemical Variables**			
Glycemia-mg/dL	96.8 ± 9.6	97.1 ± 16.5	0.605
HOMA-IR	3.7 ± 1.7	3.6 ± 1.6	0.979
Total cholesterol-mg/dL	203.1 ± 46.7	198.0 ± 33.8	0.773
HDL-c-mg/dL	51.9 ± 7.7	45.1 ± 10.0	0.022 *
LDL-c-mg/dL	114.1 ± 38.3	125.0 ± 33.0	0.315
Triglycerides-mg/dL	104.2 ± 37.3	135.3 ± 50.4	0.028 *
CRP-mg/dL	2.4 ± 1.1	3.4 ± 3.4	0.986

Quantitative data were expressed as mean ± standard deviation and qualitative data were expressed as % (*n*). * Statistical difference between MHO and MUO groups; MHO—Metabolically Healthy Obesity; MUO—Metabolically Unhealthy Obesity; BMI—Body Mass Index; WC—Waist Circumference; SAH—Systemic Arterial Hypertension; NAFLD—Non-Alcoholic Fatty Liver Disease; HOMA-IR—Homeostasis Model Assessment-Insulin Resistance; HDL—High Density Lipoprotein-c; LDL-c—Low Density Lipoprotein; CRP—C-Reactive Protein. Adolescents were classified as MHO or MUO according to the NCEP ATP III criteria adapted to adolescents.

**Table 2:** Proportion of participants who had inadequate micronutrients status with antioxidant function in the sample of adolescents with severe obesity.

Antioxidant Micronutrient	Mean ± SD	Cut-Off Points for Inadequacy	Inadequacy-% (*n*)
Vitamin C-mg/L	1.5 ± 0.6	<4.6	91.7 (55)
β-carotene-µg/dL	35.0 ± 25.7	≤40	66.7 (40)
Selenium-µg/dL	79.8 ± 23.4	<70	36.7 (22)
Retinol-µmol/L	1.5 ± 0.6	<1.05	26.7 (16)
Zinc-µg/L	87.7 ± 24.5	<75	18.3 (11)
Vitamin E-mg/dL	10.6 ± 4.4	<0.5	10.0 (6)
